# 成人KMT2A相关急性髓系白血病的临床特征及预后分析

**DOI:** 10.3760/cma.j.cn121090-20250526-00247

**Published:** 2026-04

**Authors:** 湘粤 周, 欣儿 韦, 吟 刘, 欣 孔, 晶晶 韩, 谈真 王, 一凡 沈, 剑 张, 胜利 薛, 苏宁 陈, 晓文 唐, 志洪 林, 君 陈, 惠英 仇

**Affiliations:** 1 苏州大学附属第一医院血液内科，江苏省血液研究所，国家血液系统疾病临床医学研究中心，苏州 215006 The First Affiliated Hospital of Soochow University, Jiangsu Institute of Hematology, National Clinical Research Center for Hematologic Diseases, Suzhou 215006, China; 2 苏州永鼎医院，苏州 215200 Hygeia Suzhou Yongding Hospital, Suzhou 215200, China

**Keywords:** KMT2A重排, 白血病，髓系，急性, KMT2A部分串联重复, EVI1过表达, 异基因造血干细胞移植, KMT2A-rearranged, Leukemia, myeloid, acute, KMT2A-PTD, EVI1 overexpression, Allogeneic hematopoietic stem cell transplantation

## Abstract

**目的:**

探讨成人KMT2A相关急性髓系白血病（AML）的临床生物学特征及不同亚型对预后的影响。

**方法:**

回顾性分析2014年1月至2024年8月期间于苏州大学附属第一医院就诊的303例成人KMT2A相关AML患者临床资料，分析不同伴侣基因亚组的临床生物学特征及预后差异。

**结果:**

303例KMT2A相关AML患者，中位年龄41（16～82）岁。KMT2A重排（KMT2Ar）AML占81.8％，KMT2A部分串联重复（KMT2A-PTD）AML占18.2％。KMT2Ar AML患者1个疗程完全缓解（CR）率为65.3％（162/248），2年总生存（OS）率及累积复发率（CIR）分别为52.6％（95％*CI*：46.3％～59.7％）、55.4％（95％*CI*：44.8％～61.6％）。各伴侣基因亚型CIR存在差异，KMT2A::AFDN亚型（69例）2年CIR最高，为68.5％（95％*CI*：55.4％～78.5％）；不常见KMT2Ar亚型（31例）2年CIR率最低，为43.2％（95％*CI*：24.8％～60.3％）。伴EVI1过表达的KMT2A::MLLT3患者（18例）2年CIR为66.7％（95％*CI*：38.5％～84.2％），较不伴EVI1过表达的KMT2A::MLLT3患者（53例）的44.0％（95％*CI*：30.1％～57.0％）显著升高（*P*＝0.018）。KMT2A-PTD患者中，1个疗程CR率为60.0％（33/55）；维奈克拉靶向治疗和非靶向治疗患者CR率分别为86.7％（26/30）和28.0％（7/25）（*P*<0.001），2年OS率分别为68.7％（95％*CI*：36.1％～87.1％）和53.9％（95％*CI*：32.2％～71.4％）（*P*＝0.035），2年CIR分别为34.2％（95％*CI*：15.9％～53.5％）和78.7％（95％*CI*：54.2％～91.0％）（*P*＝0.002）；强化疗和减低强度化疗患者CR率分别为40.7％（11/27）和78.6％（22/28）（*P*＝0.004），2年CIR分别为72.3％（95％*CI*：47.6％～86.8％）和41.7％（95％*CI*：20.6％～61.6％）（*P*＝0.017）。移植患者中，首次CR（CR_1_）移植（156例）和非CR_1_移植患者（62例）2年OS率分别为74.1％（95％*CI*：66.9％～82.0％）和52.0％（95％*CI*：40.6％～66.7％），2年CIR分别为27.3％（95％*CI*：20.2％～34.9％）和91.9％（95％*CI*：80.8％～96.7％）（均*P*<0.001）。Cox多因素分析示1个疗程达CR（*P*<0.001，*HR*＝0.54；*P*＝0.010，*HR*＝0.67）及异基因造血干细胞移植（*P*<0.001，*HR*＝0.17；*P*<0.001，*HR*＝0.41）是影响OS和CIR的有利因素，KMT2A::AFDN是影响OS（*P*＝0.029，*HR*＝1.51）和CIR（*P*＝0.006，*HR*＝1.56）的危险因素。

**结论:**

KMT2Ar AML中，KMT2A::AFDN亚型预后不良，伴EVI1过表达显著增加KMT2A::MLLT3患者复发风险；CR_1_移植仍是改善预后的首选策略。KMT2A-PTD对传统强化疗反应不佳，经维奈克拉靶向治疗预后显著改善。

## Abstract (en)

**Objective:**

To explore the clinicobiological characteristics and prognostic impact of different fusion partners of KMT2A in acute myeloid leukemia（AML）.

**Methods:**

The clinical features of 303 patients with KMT2A-associated AML who received treatment between January 2014 and August 2024 were retrospectively analyzed to investigate prognostic differences among fusion partner subgroups.

**Results:**

Among 303 patients with KMT2A-associated AML, the median age was 41（16–82）years. KMT2A-PTD accounted for 18.2％（55/303）, whereas KMT2A rearrangements（KMT2Ar）were observed in 81.8％（248/303）. Among KMT2Ar AML patients, the complete remission（CR）rate after a course of induction treatment was 65.3％（162/248）, whereas the 2-year overall survival（OS）rate and cumulative incidence of relapse（CIR）were 52.6％（95％*CI*: 46.3％–59.7％）and 55.4％（95％*CI*: 44.8％–61.6％）, respectively. Prognoses varied across partner gene subtypes; the KMT2A::AFDN subgroup（*n*＝69）had the poorest 2-year CIR of 68.5％（95％*CI*: 55.4％–78.5％）, whereas the uncommon KMT2Ar subgroup（*n*＝31）had a 2-year CIR rate of 43.2％（95％*CI*: 24.8％–60.3％）. The group with KMT2A::MLLT3 and EVI1 overexpression（*n*＝18）exhibited a significantly higher 2-year CIR, at 66.7％（95％*CI*: 38.5％–84.2％）versus 44.0％（95％*CI*: 30.1％–57.0％）for those with KMT2A::MLLT3 who did not show EVI1 overexpression（*n*＝53）（*P*＝0.018）. Among patients with KMT2A-PTD, the CR rate after a course of treatment was 60.0％（33/55）; venetoclax-based therapy yielded a significantly higher CR rate compared with nontargeted therapy: 86.7％（26/30）*vs* 28.0％（7/25）（*P*<0.001）, with a 2-year OS rate of 68.7％（95％*CI*: 36.1％–87.1％）versus 53.9％（95％*CI*: 32.2％–71.4％）（*P*＝0.035）, and 2-year CIR rate of 34.2％（95％*CI*: 15.9％–53.5％）versus 78.7％（95％*CI*: 54.2％–91.0％）（*P*＝0.002）. Compared to low-intensity chemotherapy, intensive chemotherapy resulted in a significantly lower CR rate: 40.7％（11/27）versus 78.6％（22/28）（*P*＝0.004）, accompanied by a higher 2-year CIR［72.3％（95％*CI*: 47.6％–86.8％）versus 41.7％（95％*CI*: 20.6％–61.6％）, *P*＝0.017］. Among transplant patients, those undergoing hematopoietic stem cell transplantation（HSCT）in CR1（*n*＝156）had better outcomes than those transplanted in non-CR1（*n*＝62）, with a 2-year OS rate of 74.1％（95％*CI*: 66.9％–82.0％）versus 52.0％（95％*CI*: 40.6％–66.7％）and 2-year CIR of 27.3％（95％*CI*: 20.0％–34.9％）versus 91.9％（95％*CI*: 80.8％–96.7％）, respectively（both *P*<0.001）. Multivariate Cox regression analysis showed that achieving CR after one induction cycle（*HR*＝0.54, *P*<0.001; *HR*＝0.67, *P*＝0.010）and receiving allo-HSCT（*HR*＝0.17, *P*<0.001; *HR*＝0.41, *P*<0.001）were favorable prognostic factors for OS and CIR. KMT2A::AFDN was an adverse prognostic factor for both OS（*HR*＝1.51, *P*＝0.029）and CIR（*HR*＝1.56, *P*＝0.006）.

**Conclusion:**

In KMT2Ar AML, the KMT2A::AFDN subtype was associated with adverse prognosis, whereas concomitant EVI1 overexpression significantly increased the risk of relapse in KMT2A::MLLT3 subgroup. allo-HSCT in CR_1_ significantly improves prognosis in these patients. The KMT2A-PTD group showed poor response to conventional intensive chemotherapy but achieved significantly improved prognosis with venetoclax-based therapy.

KMT2A相关白血病源于KMT2A基因内部发生断裂引起染色体相互易位或者N端内部串联重复序列插入，继而引起HOX基因簇及MEIS1异常过表达，最终导致造血分化阻滞和白血病转化[1]–[2]，占成人急性髓系白血病（AML）患者的5％～10％，其伴侣基因广泛，目前已发现高达100种以上[3]。不同伴侣基因之间预后存在差异，目前关于不同伴侣基因与预后之间的相关性仍存在争议[4]。KMT2A部分串联重复（KMT2A-PTD）作为KMT2A相关AML中的一种特殊类型，常规细胞遗传学检测不容易发现，既往报道很少，近年来随着转录组靶向测序（RNA-seq）等检测技术的应用其检出率明显提高，但尚没有划定明确的预后危险分层标准[5]–[7]。本研究分析了303例KMT2A相关AML患者的临床资料，以探究不同亚型患者的临床特征及预后差异。

## 病例与方法

1. 病例：回顾性收集了2014年1月至2024年8月期间在苏州大学附属第一医院接受治疗的312例成人KMT2A相关AML患者临床资料。根据2022版WHO造血和淋巴组织肿瘤的分型诊断标准对所有患者进行重新诊断及分型，且通过实时聚合酶链反应（RT-PCR）、荧光原位杂交（FISH）、二代测序（NGS）或者RNA-seq等技术检出KMT2A重排（KMT2Ar）或KMT2A-PTD。排除4例未经治疗患者（2例为KMT2A::MLLT3、1例为KMT2A::AFDN、1例为KMT2A-PTD），5例无法评估疗效患者（2例为KMT2A::MLLT3、2例为KMT2A::ELL、1例为KMT2A::AFDN），本次研究纳入303例患者。

2. 治疗方案：强化化疗：①IA或DA（7+3方案）：去甲氧柔红霉素8～12 mg/m^2^或柔红霉素45～90 mg/m^2^第1～3天，联合阿糖胞苷100 mg/m^2^第1～7天；②HAA方案：高三尖杉酯碱1～2 mg/m^2^第1～7天，阿糖胞苷100～200 mg/m^2^第1～7天，阿克拉霉素20 mg/d第1～7天；③维奈克拉联合IA或HAA方案。

低强度化疗：①维奈克拉联合去甲基化药物（地西他滨或阿扎胞苷）：地西他滨20 mg/m^2^第1～5天或阿扎胞苷75 mg/m^2^第1～7天；②预激方案：去甲氧柔红霉素5 mg/m^2^第1、3、5天，或阿克拉霉素20 mg/d第1～4天，联合阿糖胞苷10 mg/m^2^第1～14天，G-CSF 150 µg/d第0～14天（根据血象调整）；③地西他滨联合预激方案。维奈克拉采用100 mg第1天，200 mg第2天，400 mg第3～28天爬坡（根据血象及药物相互作用进行调整）。化疗方案选择根据患者年龄、脏器功能评估以及主治医师经验综合决定。

诱导治疗达缓解后采用原方案、中高剂量阿糖胞苷或造血干细胞移植巩固治疗。移植预处理方案均为改良BuCy方案（具体为：司莫司汀250 mg·m^−2^·d^−1^，−10 d；阿糖胞苷4 g·m^−2^·d^−1^，−9 d至−8 d；白消安3.2 mg·kg^−1^·d^−1^，−7 d至−5 d；环磷酰胺1.8 g·m^−2^·d^−1^，−4 d至−3 d）。

3. 疗效评估及定义：完全缓解（CR）：骨髓原始细胞<5％，无外周或髓外原始细胞浸润，中性粒细胞≥1×10^9^/L，PLT≥100×10^9^/L。复发：骨髓原始细胞≥5％或出现髓外浸润。WT1过表达：WT1转录本/ABL拷贝数>2％；EVI1过表达：EVI1转录本/ABL拷贝数>10％。总生存（OS）期定义为诱导治疗开始到因任何原因死亡或失访或末次随访时间。

4. 随访：通过查阅住院病历、门诊资料以及电话方式随访，随访截止时间2025年3月19日。5例（1.7％）患者失访。

5. 统计学处理：采用SPSS21.0以及R4.4.2软件进行统计分析及绘图。计数资料以频数和百分率（％）描述，计量资料以中位数（范围）来描述，分类变量组间比较采用卡方检验或Fisher确切概率法，两组连续变量比较使用Mann-Whitney *U*检验，多组连续变量比较采用Kruskal-Wallis *H*检验。通过Kaplan-Meier法绘制OS曲线，Log-rank检验比较组间差异。累积复发率（CIR）采用竞争风险模型分析，非复发死亡为竞争事件，并通过Gray检验比较差异。使用单因素及多因素Cox分析评估疾病预后影响因素。双侧检验*P*<0.05为差异有统计学意义。

## 结果

一、临床特征及亚型分类

KMT2A相关AML患者初诊中位年龄为41（16～82）岁，男性患者占47.5％，中位初诊WBC为20.7（0.5～350.8）×10^9^/L，51.2％患者为FAB-M_5_亚型，71.9％患者经异基因造血干细胞移植（allo-HSCT）治疗，其中51.5％患者在首次CR（CR_1_）期移植。KMT2Ar（248例）常见伴侣基因分别为KMT2A::MLLT3（28.6％）、KMT2A::AFDN（27.8％）、KMT2A::ELL（21.8％）、KMT2A::MLLT10（9.3％）；其余各伴侣基因比例均不超过4％，统称为不常见KMT2Ar亚组，占12.5％。55例（18.2％）患者涉及KMT2A-PTD，因发病机制不同于KMT2Ar患者被单独列出。KMT2Ar各伴侣基因以及KMT2A-PTD亚组间在年龄、WBC、骨髓原始细胞比例、FAB-M_5_亚型以及染色体核型方面存在差异。其中KMT2A::MLLT3与KMT2A::MLLT10亚组年龄最小（中位年龄均为32岁），而KMT2A-PTD亚组中位年龄最大（48岁）。KMT2A::MLLT3与KMT2A-PTD亚组初诊中位WBC较低。在KMT2A-PTD亚组中，18.2％患者为FAB-M_5_亚型，72.7％为正常染色体核型（表1）。

**表1:** 303例KMT2A相关急性髓系白血病患者的临床特征

特征	所有患者（303例）	KMT2A::MLLT3（71例）	KMT2A::AFDN（69例）	KMT2A-PTD（55例）	KMT2A::ELL（54例）	KMT2A::MLLT10（23例）	不常见KMT2Ar（31例）	*P*值
年龄［岁，*M*（范围）］	41（16～82）	32（16～65）	39（16～82）	48（19～67）	45（17～67）	32（16～63）	37（20～63）	0.001
男性［例（％）］	144（47.5）	35（49.3）	33（47.8）	29（52.7）	25（46.3）	14（60.9）	8（25.8）	0.144
WBC［×10^9^/L,*M*（范围）］	20.7（0.5～350.8）	8.8（0.5～324.0）	44.5（1.3～326.7）	3.4（0.5～220.8）	20.2（0.5～350.8）	35.2（1.2～140.7）	40.2（0.9～185.7）	<0.001
骨髓原始细胞［％，*M*（范围）］	78.0（20.5～99.5）	86.4（23.0～99.5）	80.0（27.5～98.0）	61.0（20.5～98.0）	67.5（20.5～93.0）	89.4（38.0～96.0）	81.0（25.2～96.0）	<0.001
FAB-M_5_亚型［例（％）］	155（51.2）	44（62.0）	45（65.2）	10（18.2）	18（33.3）	18（78.3）	20（64.5）	<0.001
额外细胞遗传学异常［例（％）］	87（28.7）	19（26.8）	19（27.5）	15（27.3）	13（24.1）	10（43.5）	11（35.5）	0.718
正常核型［例（％）］	111（36.6）	25（35.2）	25（36.2）	40（72.7）	8（14.8）	7（30.4）	6（19.4）	<0.001
复杂核型［例（％）］	26（8.6）	5（7.0）	6（8.7）	1（1.8）	5（9.3）	5（21.7）	4（12.9）	0.256
三体8［例（％）］	27（8.9）	9（12.7）	4（5.8）	2（3.6）	3（5.6）	4（17.4）	5（16.1）	0.301
诱导治疗［例（％）］								
强化疗	180（59.4）	48（67.6）	48（69.6）	27（49.1）	30（55.6）	14（60.9）	13（41.9）	0.040
减低强度化疗	123（40.6）	23（32.4）	21（30.4）	28（50.9）	24（44.4）	9（39.1）	18（58.1）	
维奈克拉靶向治疗	109（36.0）	18（25.4）	21（30.4）	30（54.5）	16（29.4）	10（43.5）	14（45.2）	0.009
移植［例（％）］	218（71.9）	60（84.5）	43（62.3）	38（69.1）	39（72.2）	15（65.2）	23（74.2）	0.089
CR_1_移植［例（％）］	156（51.5）	40（56.3）	30（43.4）	27（49.0）	32（59.3）	9（39.1）	18（58.1）	0.325

**注** 额外细胞遗传学异常：除KMT2A染色体融合易位之外的染色体结构或数目异常；KMT2Ar：KMT2A重排；CR_1_：首次诱导完全缓解

二、伴随突变情况

303例KMT2A相关AML患者中，287例有明确染色体核型信息，其中147例涉及11q23核型异常，111例为正常核型，87例存在额外细胞遗传学异常，其中三体8核型占8.9％（27/303）。263例患者通过NGS检测51种常见造血系统基因突变，共发现44种伴随基因突变（图1）；KMT2Ar AML常见的伴随突变为KRAS（26.3％）、NRAS（21.5％）、FLT3-ITD（10.5％）、STAG2（8.1％）、PTPN11（9.0％）、GATA2（7.1％）、WT1（7.1％）等，KMT2Ar AML患者不同伴侣基因亚组之间伴随突变相似，而KMT2A-PTD亚组以FLT3-ITD（29.6％）、DNMT3A（27.7％）、CEBPA（14.8％）等伴随突变最常见。95例（31.3％）KMT2A相关AML患者初诊时合并WT1过表达，73例（24.1％）患者合并EVI1过表达，KMT2A-PTD亚组均合并EVI1低表达。

**图1:**
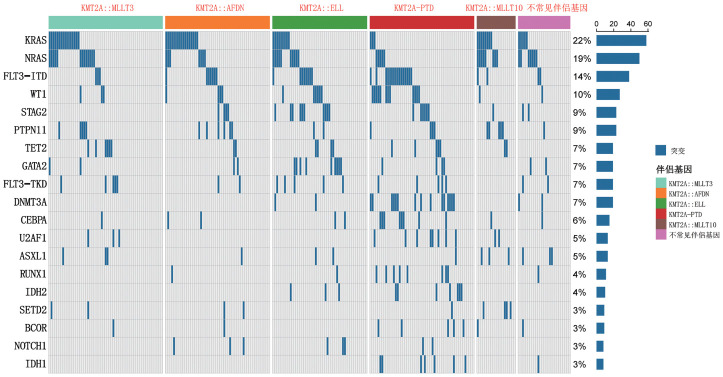
KMT2A相关急性髓系白血病伴随基因突变图谱（仅展示突变频率大于3％的基因）

三、疗效反应与预后分析

1. KMT2Ar AML患者疗效及预后：总体KMT2Ar AML患者的1个疗程CR率为65.3％，不同KMT2Ar伴侣基因亚组间CR率存在差异，但差异无统计学意义（*P*＝0.176），KMT2A::ELL亚型CR率最高，为75.9％；KMT2A::MLLT10亚型CR率最低，为52.1％（表2）。中位随访时间41.2（2.1～122.3）个月，KMT2Ar AML患者2年OS率为52.6％（95％*CI*：46.3％～59.7％）、2年CIR为55.4％（95％*CI*：44.8％～61.6％）。各伴侣基因亚组间OS差异无统计学意义（*P*＝0.235），而KMT2A::AFDN亚组复发率最高，2年CIR为68.5％（95％*CI*：55.4％～78.5％），不常见KMT2Ar亚组复发率最低，2年CIR为43.2％（95％*CI*：24.8％～60.3％）（图2）。

**表2:** KMT2A相关急性髓系白血病患者在不同治疗下的完全缓解（CR）率（％）

组别	例数	总体患者CR率	不同强度化疗					靶向治疗				
			强化疗		减低强度化疗		*P*值	维奈克拉靶向治疗		非靶向治疗		*P*值
			例数	CR率	例数	CR率		例数	CR率	例数	CR率	
KMT2A-PTD	55	60.0	27	40.7	28	78.6	0.004	30	86.7	25	28.0	<0.001
KMT2Ar	248	65.3	153	65.4	95	65.2	0.988	79	67.1	169	64.5^a^	0.690
KMT2A：：MLLT3	71	67.6	48	70.8	23	60.9	0.401	18	61.1	53	69.8	0.496
KMT2A：：AFDN	69	57.9	48	54.2	21	66.7	0.333	21	71.4	48	52.1	0.134
KMT2A：：ELL	54	75.9	30	73.3	24	79.2	0.618	16	87.5	38	71.1	0.301
KMT2A：：MLLT10	23	52.1	14	64.3	9	33.3	0.214	10	40.0	13	61.5	0.414
不常见KMT2Ar	31	67.7	13	69.2	18	66.7	0.880	14	64.3	17	70.6	0.709

**注** KMT2Ar：KMT2A重排。^a^与KMT2A-PTD组比较，*P*<0.001

**图2:**
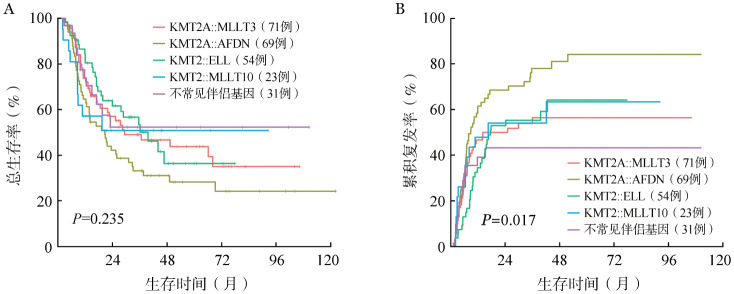
KMT2A重排急性髓系白血病各伴侣基因亚组总生存（A）及累积复发（B）曲线

2. 不同诱导治疗方案对KMT2Ar AML患者疗效和预后的影响：在KMT2Ar AML患者中，经1个疗程诱导治疗，强化疗组与减低强度化疗组CR率分别为65.4％和65.2％（*P*＝0.998），维奈克拉靶向治疗组和非维奈克拉靶向治疗组CR率分别为67.1％和64.5％（*P*＝0.690），差异均无统计学意义。KMT2Ar各伴侣基因亚组中，不同诱导方案治疗下CR率差异亦无统计学意义（表2）。强化治疗组和减低强度化疗组移植率分别为67.5％和75.0％（*P*＝0.152），维奈克拉靶向治疗组和非靶向治疗组移植率分别为71.6％和72.2％（*P*＝0.910），差异均无统计学意义。长期预后方面，强化治疗组与减低强度化疗组2年OS率分别为54.8％（95％*CI*：46.2％～62.7％）和48.4％（95％*CI*：36.6％～59.1％）（*P*＝0.171），2年CIR分别为56.5％（95％*CI*：48.1％～64.1％）和54.0％（95％*CI*：42.6％～64.1％）（*P*＝0.532）；各伴侣基因亚组间OS及CIR差异无统计学意义（**扫描本文首页二维码查阅附表1**）。维奈克拉靶向治疗组和非靶向治疗组2年OS率分别为55.4％（95％*CI*：40.9％～67.6％）和51.3％（95％*CI*：43.4％～58.7％）（*P*＝0.455），2年CIR分别为46.0％（95％*CI*：33.7％～57.5％）和59.1％（95％*CI*：51.1％～66.1％）（*P*＝0.158），差异无统计学意义；而KMT2A::ELL亚组中，维奈克拉治疗倾向于改善OS及CIR（*P*值分别为0.064、0.087）。其余伴侣基因亚组间OS及CIR差异无统计学意义（**扫描本文首页二维码查阅附表1**）。

3. KMT2A-PTD患者的疗效及预后：经1个疗程诱导治疗，KMT2A-PTD患者CR率为60.0％，其中维奈克拉靶向治疗组CR率显著高于非靶向治疗组（86.7％对28.0％，*P*<0.001）；强化疗组CR率显著低于减低强度化疗组（40.7％对78.6％，*P*＝0.004）。维奈克拉靶向治疗组和非靶向治疗组移植率分别为70.0％和68.0％（*P*＝0.873），强化疗组和减低强度化疗组移植率分别为64.3％和74.1％（*P*＝0.432），差异均无统计学意义。中位随访21.9（2.9～68.4）个月，KMT2A-PTD患者2年OS率及CIR分别为63.3％（95％*CI*：49.8％～80.4％）、57.9％（95％*CI*：41.2％～71.3％）；维奈克拉靶向治疗组2年OS率、CIR明显优于非靶向治疗组［68.7％（95％*CI*：36.1％～87.1％）对53.9％（95％*CI*：32.2％～71.4％），*P*＝0.035；34.2％（95％*CI*：15.9％～53.5％）对78.7％（95％*CI*：54.2％～91.0％），*P*＝0.002］。强化疗组2年CIR显著高于减低强度化疗［72.3％（95％*CI*：47.6％～86.8％）对41.7％（95％*CI*：20.6％～61.6％），*P*＝0.017］。

4. 合并WT1过表达及EVI1过表达患者预后：合并WT1过表达患者和非WT1过表达患者2年OS率分别为39.4％（95％*CI*：30.6％～50.9％）和62.6％（95％*CI*：55.7％～70.4％）（*P*<0.001），2年CIR分别为68.2％（95％*CI*：57.6％～76.7％）和49.8％（95％*CI*：42.3％～56.7％）（*P*<0.001）。在KMT2A::MLLT3、其他非KMT2A::MLLT3的KMT2Ar患者中伴WT1过表达较不伴WT1过表达患者OS率低、CIR高；在KMT2A-PTD患者中，伴WT1过表达较不伴WT1过表达患者CIR高（图3）。合并EVI1过表达的KMT2A::MLLT3亚型患者相较未合并EVI1过表达患者预后差：2年OS率分别为50.0％（95％*CI*：31.5％～79.4％）和59.9％（95％*CI*：47.2％～76.0％）（*P*＝0.023），2年CIR分别为66.7％（95％*CI*：38.5％～84.2％）和44.0％（95％*CI*：30.1％～57.0％）（*P*＝0.018）；而伴或不伴EVI1过表达在非KMT2A::MLLT3重排患者中预后差异无统计学意义（图4）。

**图3:**
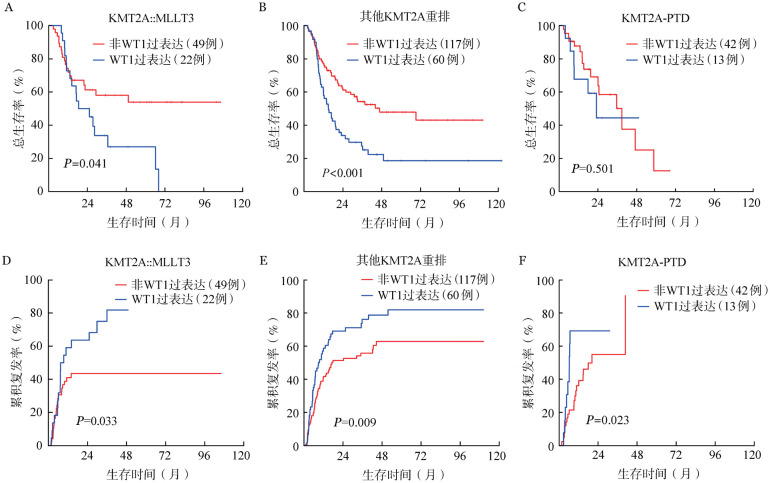
KMT2A::MLLT3、其他KMT2A重排以及KMT2A-PTD患者伴与不伴WT1过表达组的总生存（A、B、C）和累积复发（D、E、F）曲线比较

**图4:**
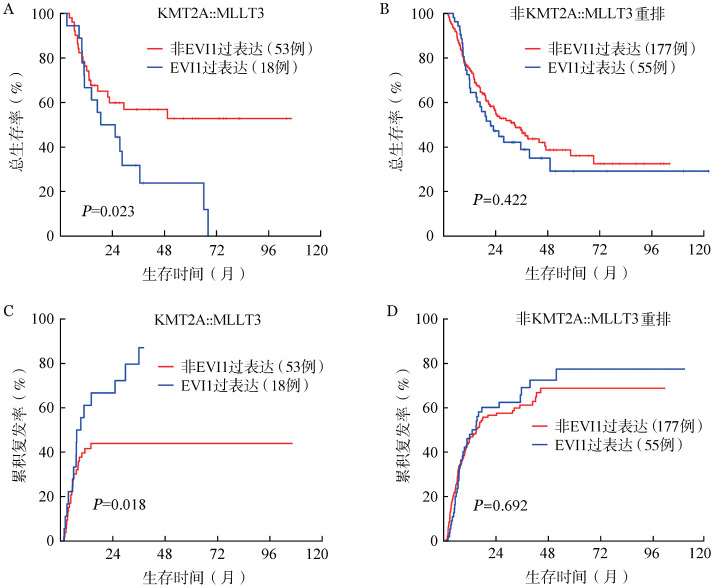
KMT2A::MLLT3、非KMT2A::MLLT3重排患者伴与不伴EVI1过表达组的总生存（A、B）及累积复发（C、D）曲线

5. allo-HSCT对KMT2A相关AML患者预后的影响：218例KMT2A相关AML后续接受allo-HSCT治疗，其中156例为CR_1_期移植，27例为CR_2_/CR_n_阶段移植，35例挽救性移植。移植与非移植患者相比OS期得到延长，总体CIR降低；2年OS率及CIR分别为67.3％（95％*CI*：60.9％～74.4％）对15.9％（95％*CI*：8.8％～28.8％）、46.0％（95％*CI*：39.0％～52.7％）对83.2％（95％*CI*：71.2％～90.5％）（均*P*<0.001）。其中CR_1_移植组和非CR_1_移植组相比远期预后明显改善：2年OS率及CIR率分别为74.1％（95％*CI*：66.9％～82.0％）对52.0％（95％*CI*：40.6％～66.7％）、27.3％（95％*CI*：20.2％～34.9％）对91.9％（95％*CI*：80.8％～96.7％）（均*P*<0.001）（图5）。

**图5:**
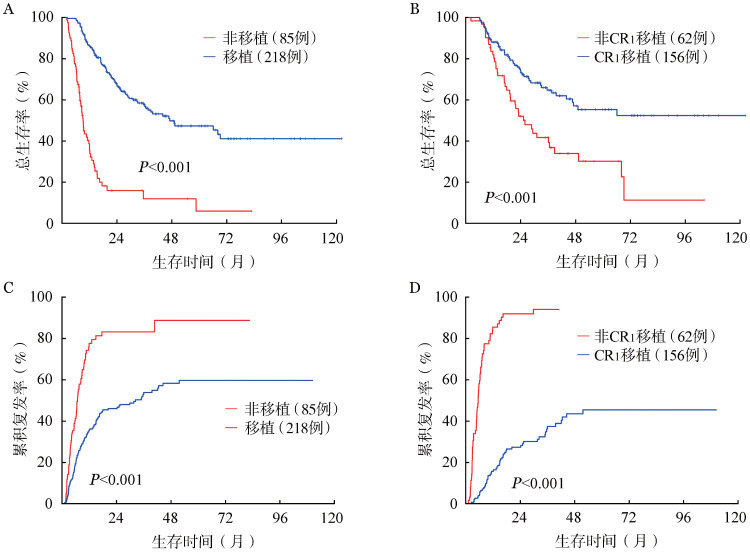
移植对成人KMT2A相关急性髓系白血病患者生存的影响 **A、C** 移植与非移植患者的总生存和累积复发曲线；**B、D** 首次完全缓解（CR_1_）移植与非CR_1_移植患者的总生存和累积复发曲线

6. 预后影响因素分析：对KMT2A相关AML患者进行预后单因素分析，纳入因素包括年龄>60岁、高白细胞（>100×10^9^/L）、FAB-M_5_亚型、继发/治疗相关AML、额外细胞遗传学异常、复杂核型、三体8、非伴侣基因（KMT2A-PTD）、伴侣基因（KMT2A::MLLT3、KMT2A::AFDN、KMT2A::ELL、KMT2A::MLLT10、不常见KMT2Ar）、1个疗程诱导治疗CR、强化疗、靶向治疗、allo-HSCT，将*P*<0.1的预后因素纳入多因素分析，结果显示：经过1个疗程诱导治疗CR以及allo-HSCT是影响患者OS及CIR的有利因素。KMT2A::AFDN是影响患者OS及CIR的危险因素，而复杂核型是影响CIR的危险因素（表3）。

**表3:** KMT2A相关急性髓系白血病（AML）患者OS及CIR预后的多因素分析

因素	OS		CIR	
	*HR*（95％*CI*）	*P*值	*HR*（95％*CI*）	*P*值
年龄>60岁	0.99（0.55～1.76）	0.962	1.41（0.93～2.16）	0.109
复杂核型	1.44（0.87～2.39）	0.154	2.05（1.19～3.51）	0.009
KMT2A::AFDN	1.51（1.04～2.17）	0.029	1.56（1.14～2.13）	0.006
1个疗程诱导治疗CR	0.54（0.39～0.76）	<0.001	0.67（0.49～0.91）	0.010
强化疗	–	–	1.09（0.76～1.56）	0.643
靶向治疗	0.83（0.55～1.26）	0.384	0.71（0.46～1.09）	0.121
allo-HSCT	0.17（0.12～0.24）	<0.001	0.41（0.29～0.58）	<0.001

**注** OS：总生存；CIR：累积复发率；CR：完全缓解；allo-HSCT：异基因造血干细胞移植；–：未纳入多因素分析

## 讨论

本研究KMT2Ar AML患者中，伴侣基因组成以KMT2A::MLLT3（28.6％）、KMT2A::AFDN（27.8％）、KMT2A::ELL（21.8％）、KMT2A::MLLT10（9.3％）多见，与既往研究相类似[8]。而KMT2A-PTD患者占18.2％，高于既往文献报道的5％～10％[3],[9]，这种差异可能源于检测方法的不断更新，本研究中52.7％（29/55）KMT2A-PTD患者经RNA-seq检测确诊。多项研究表明KMT2A-PTD与KMT2Ar AML临床特征存在差异[7],[10]。本研究中KMT2A-PTD亚型患者初诊年龄较大（中位年龄48岁），WBC最低（中位3.4×10⁹/L），72.7％表现为正常核型，且FAB-M_5_亚型比例低；伴随突变以表观遗传调控基因突变（DNMT3A 27.7％，CEBPA 14.8％）和FLT3-ITD（29.6％）为主。相比之下，KMT2Ar AML患者以RAS通路激活（KRAS/NRAS）和FLT3-ITD突变为主，且多伴单核细胞分化。而KMT2Ar伴侣基因亚型间也存在差异，其中KMT2A::AFDN亚组白细胞计数最高，KMT2A::MLLT10亚组骨髓原始细胞比例最高且M_5_表型最多，KMT2A::ELL亚型单核表型比例较低。

KMT2Ar AML患者中伴侣基因种类对预后有明显影响，多项研究均证实在KMT2A相关AML中KMT2A::AFDN预后最差，具有生存期短且易短期复发特点[11]–[13]。国外一项研究中21例KMT2A::AFDN AML患者的3年OS率仅5％，3年无病生存（DFS）率为0[12]。本研究中KMT2A各伴侣基因间KMT2A::AFDN亚型仍预后最差，2年OS率和2年CIR率分别为42.2％和68.5％。OS优于国外报道，可能与我中心移植率高（43/69）有关。目前KMT2A::MLLT3作为最常见的伴侣基因亚型之一，被纳入2022ELN指南的中危预后组，但仍有部分研究认为KMT2A::MLLT3患者预后与其余KMT2Ar AML患者无明显差异[8],[14]。Bill等[12]在对172例11q23/KMT2Ar AML患者的回顾性研究中，认为年轻（<60岁）KMT2A::MLLT3患者预后要优于其他11q23/KMT2Ar AML患者，而老年（>60岁）KMT2A::MLLT3 AML患者预后较差。然而在我们的研究中，98.6％（70/71）KMT2A::MLLT3患者小于60岁，但KMT2A::MLLT3患者相较其他伴侣基因并无明显生存获益。我们发现EVI1过表达对KMT2A::MLLT3亚型AML患者预后具有显著负面影响，使复发进程加快且OS期明显缩短；鉴于其显著增加的复发风险及不良预后，我们建议这类患者在首次缓解后尽早行allo-HSCT，以期最大限度降低复发率并改善远期生存。值得注意的是，这种预后差异在非KMT2A::MLLT3重排患者中未观察到。本研究中EVI1过表达阈值10％，KMT2A-PTD亚组中均合并EVI1低表达。而另一项国内单中心研究设置EVI1过表达阈值8％，86例KMT2A-PTD患者中仅检出4例（4.7％）伴EVI1过表达[15]。尽管本研究及目前有限的文献均观察到了这种现象，但尚未对其背后的机制进行深入探讨。目前尚不明确这种低表达是否反映了潜在的生物学互斥关系。要揭示其机制，仍需多中心更大样本量数据以及更深入的分子生物学研究来证实。

本研究在对于不同治疗方案下疗效及预后分析发现，KMT2A-PTD在不同治疗方案下展现出明显疗效及预后差异，该亚型对传统强化疗反应不佳，CR率仅为40.7％，2年CIR高达72.3％；而对于维奈克拉靶向治疗较敏感（CR率86.7％），总生存与无复发生存均获显著改善。但KMT2Ar AML在不同治疗方案下并未显示有统计学差异的预后结局。这也进一步证实KMT2A-PTD与KMT2Ar AML患者具有显著临床异质性，致病机制也可能不同于KMT2Ar经典的融合驱动途径。KMT2A-PTD患者富含的表观遗传学突变以及低单核表达可能共同导致了该亚型对于维奈克拉靶向治疗的特殊敏感性。在当前多数menin抑制剂临床试验尚未系统报道KMT2A-PTD患者的背景下，以维奈克拉为基础的靶向治疗方案有望成为该群体诱导治疗的重要选择，从而改善其整体预后。目前移植仍是KMT2A相关AML患者的首要选择[13]，但本中心统计的KMT2A相关AML移植2年内仍有46.0％的复发率，其中CR_1_移植能延缓患者的复发进程，2年的复发率下降至27.3％；这表明在CR_1_期尽早进行移植是实现最佳生存获益的关键时机。针对如何提高这部分患者缓解率以及延缓复发，新的靶向及免疫治疗研究同样在进展中，关于menin抑制剂revumenib的Ⅱ期临床试验显示，在复发/难治性KMT2A重排AML中总反应率为63.2％，CR+CRh率为22.8％[16]。另一项关于抗CD33单抗（GO单抗）治疗儿童KMT2Ar AML的Ⅲ期临床试验中，GO组复发率较非GO组显著降低（40％对66％），且HSCT前使用GO单抗可进一步减少移植后复发（GO组28％对非GO组73％）[17]。但目前GO在成人KMT2Ar AML中的研究数据仍较有限，仍需更多临床研究验证其疗效和安全性。

本研究尚存一定局限性：作为单中心回顾性研究，可能存在选择偏倚；不同检测技术的选择可能导致相关伴侣基因检出率存在差异。本研究结果显示：对于KMT2Ar AML而言，KMT2A::AFDN亚型仍与不良预后相关。而伴EVI1过表达使KMT2A::MLLT3生存期缩短，复发率显著提高，提示其作为该亚型精细风险分层的新标志物价值。KMT2A-PTD亚型对传统强化疗反应不佳，在维奈克拉为基础的靶向治疗下缓解率提高及预后改善。

## Supplementary Material


