# Supplementary figures and images for: Clinical characteristics and prognostic analysis of adult KMT2A-associated acute myeloid leukemia
Source: Zhonghua Xue Ye Xue Za Zhi. 2026 Apr;47(4):336–44. [Article in Chinese] doi: 10.3760/cma.j.cn121090-20250526-00247 (PMC13195550; doi:10.3760/cma.j.cn121090-20250526-00247)

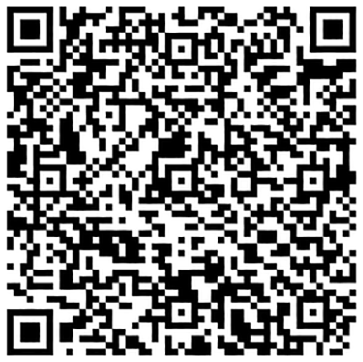

Supplement: Supplementary file 1 [file cjh-47-04-336-g006.tif]
